
JOURNAL INFORMATION
==============================
NLM Title Abbreviation: ScientificWorldJournal
Journal ID: ScientificWorldJournal
NLM Journal ID: 101131163
Journal ID: TSWJ

The Scientific World Journal

ISSN: 2356-6140
EISSN: 1537-744X
Publisher: Wiley

ARTICLE INFORMATION
==============================
PMCID: PMC6084137
PMID: 30147575
DOI: 10.1100/tsw.2001.202
Article ID: 457976
Article version: 1
Subjects: Short Report

African Swine Fever Virus (Asfv) Multigene Families 360 and 530 Genes Promote Infected Macrophage Survival

Zsak L. *
Sur J.H.
Burrage T.G.
Neilan J.G.
Rock D.L.
Plum Island Animal Disease Center, USDA, ARS, P.O. Box 848, Greenport, NY 11944, USA
*L. Zsak: lzsak@piadc.ars.usda.gov
Publication date: 2001
Electronic publication date: 2001 Dec 8
Volume: 1
First page: 97
Last page: 97
Copyright: Copyright © 2001 L. Zsak et al.
Copyright year: 2001
License: This is an open access article distributed under the Creative Commons Attribution License, which permits unrestricted use, distribution, and reproduction in any medium, provided the original work is properly cited.
License URL: https://creativecommons.org/licenses/by/3.0/

==============================
Miami *Nature Biotechnology* Short Reports
*TheScientificWorld* (2001) 1 (S3), 97SR
ISSN 1532-2246; DOI 10.1100/tsw.2001.202

### AFRICAN SWINE FEVER VIRUS (ASFV) MULTIGENE FAMILIES 360 AND 530 GENES PROMOTE INFECTED MACROPHAGE SURVIVAL

L. Zsak*, J.H. Sur, T.G. Burrage, J.G. Neilan, and D.L. Rock

Plum Island Animal Disease Center, USDA, ARS, P.O. Box 848, Greenport, NY 11944
* lzsak@piadc.ars.usda.gov

**INTRODUCTION.** In the swine host ASFV infects cells of the mononuclear-phagocytic system including fixed tissue macrophages (1). Cytopathological changes consistent with apoptotic cell death, including karyorrhexis and chromatin condensation, have been observed in mononuclear cells in tissues of pigs infected with highly virulent ASFV isolates (2). ASFV infection induced apoptosis in primary swine macrophages *in vitro* at late time postinfection, a time at which viral replication had already occurred in these cells (3). Thus, transient modulation of infected macrophage survival may be necessary for productive viral replication to occur in this cell type.

**METHOD.** ASF viruses were grown in primary macrophage cell cultures as previously described (3). Trypan blue dye exclusion assay was used to determine cell viability; TUNEL assay and Cell Death Detection ELISA $^{plus}$ kit were applied to detect apoptosis in infected cells.

**RESULTS.** In marker rescue and reverse genetics experiments we found that deletion of a 10-kbp region from the left variable terminus of pathogenic ASFV genomes, containing six multigene family (MGF) 360 and two MGF 530 genes, markedly reduced viral growth in swine macrophage cell cultures. Cell viability studies and apoptosis assays revealed a significant reduction in survival time, and accelerated and increased apoptotic cell death in gene deletion mutant, Δ35-infected macrophage cell cultures (see Fig. 1).

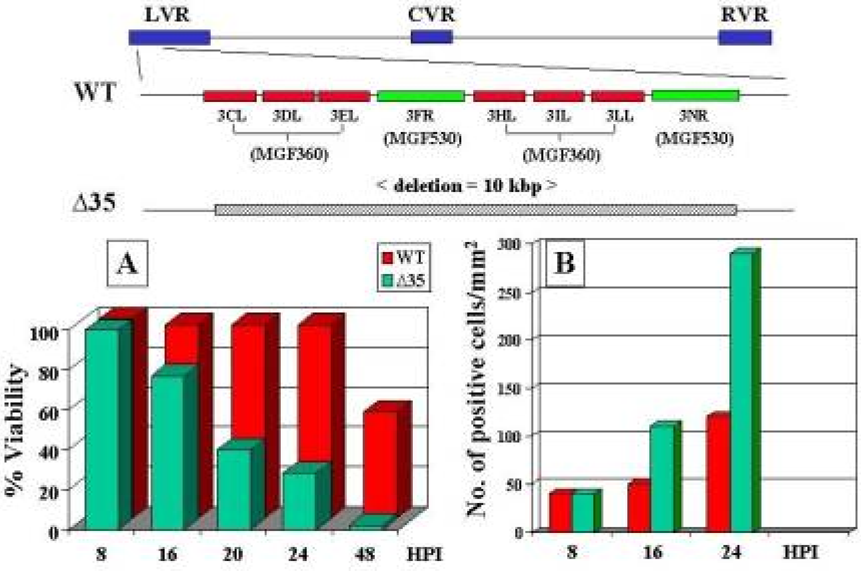

To examine gene function(s) in swine we have compared the pathogenesis of Δ35 infection to its revertant virus, Δ35-R. In contrast to Δ35-R-infected pigs, where 100% of the animals died within 10 days postinfection, all Δ35-infected pigs survived infection. Virus titers in lymphoid tissues, lung, and liver were significantly reduced by 100-10,000-fold over revertant virus values. Immunohistochemistry showed a markedly reduced number of Δ35-infected cells in those tissues; however, a significantly increased rate of apoptotic cell death was observed when compared to Δ35-R at 3 days postinfection.

**DISCUSSION.** Our data indicate that ASFV MGF 360 and MGF 530 genes perform an essential macrophage host range function(s) that involves the promotion of infected cell survival. Reduced viral growth in macrophages together with increased early apoptotic death of virus-infected cells likely account for the attenuation of Δ35 in swine.

**ACKNOWLEDGEMENT.** We thank Aniko Zsak and the PIADC animal care staff for excellent technical assistance.

**REFERENCES.**

1. Mebus, C.A. (1988) Adv. Virus Res. 35, 251-269
2. Colgrove, G.S., Haelterman, E.O., and Coggins, L. (1969) Am. J. Vet. Res. 30, 1343-1359
3. Neilan, J.G., Lu, Z., Kutish, G.F., Zsak, L., Burrage, T.G., Borca, M.V., Carrillo, C., and Rock, D.L. (1997) Virology 230, 252-264
